# Closed Incision Negative Pressure Therapy: Review of the Literature

**DOI:** 10.7759/cureus.5183

**Published:** 2019-07-21

**Authors:** Luis G Fernandez, Marc R Matthews, Pablo Sibaja Alvarez, Scott Norwood, David H Villarreal

**Affiliations:** 1 Surgery, Trauma Wound Care, University of Texas Health Science Center, Tyler, USA; 2 Surgery, Arizona Burn Center, Phoenix, USA; 3 General Surgery, Universidad Federada San Judas Tadeo, San Jose, CRI; 4 Surgery, University of Texas Health Science Center, Tyler, USA; 5 Trauma, Acute Care Surgery, Surgical Critical Care, University of Texas Health Science Center, Tyler, USA

**Keywords:** closed incision negative pressure therapy, literature reviews, health economics

## Abstract

Surgical site infection and other common surgical site complications (dehiscence, hematoma, and seroma formation) can lead to serious and often life-threatening complications. Gauze, adhesive dressings, and skin adhesives have traditionally been utilized for incision management. However, the application of negative pressure wound therapy over clean, closed surgical incisions (closed incision negative pressure therapy, ciNPT), has become a recent option for incision management. A brief review of ciNPT clinical evidence and health economic evidence are presented. A brief literature review was performed using available publication databases (PubMed, Ovid®, Embase®, and QUOSA™) for articles in English reporting on the use of ciNPT between October 1, 2016, to March 31, 2019. The successful application of ciNPT over clean, closed wounds has been reported in a broad spectrum of patients and operative interventions, resulting in favorable clinical results. Four of the five studies that examined health economics following the use of ciNPT reported a potential reduction in the cost of care. The authors’ own experience and published results suggest that patients at high risk for developing a surgical site complication may benefit from the use of ciNPT during the immediate postoperative period. Additional studies are needed across various surgical disciplines to further assess the safety, and cost-effectiveness of ciNPT use in patient populations.

## Introduction and background

Surgical site infections (SSIs) and other common surgical site complications (dehiscence, hematoma, and seroma formation) can lead to serious and often life-threatening complications. Recent reports suggest that there are 8.2 million people at risk for SSIs annually in the United States [1-3]. SSIs frequently occur and are now the most common and costly of all healthcare-acquired infections, with a reported incidence ranging from 15-37% [4-7]; and accounts for 33.7% of the $9.8 billion costs to the US healthcare system per year [1].

Standard of care (SOC) therapy typically consists of dry or moistened gauze, abdominal pads, adhesive dressings, or skin adhesives. However, gauze dressings have been criticized for their inherent nonocclusive nature [8] and associated with a higher infection rate than transparent films or hydrocolloids [9, 10]. A more recent option for surgical incision management, especially in patients at high risk of developing surgical site complications, is the use of closed incision negative pressure (ciNPT). A brief literature search is presented.

## Review

Methods

Literature Search

A literature search was performed using available publication databases (PubMed, Ovid®, Embase®, and QUOSA™) for peer-reviewed articles published between October 1, 2016, and March 31, 2019. Keywords included “Prevena”, “NPT” (negative pressure therapy), “Negative wound therapy”, and “negative pressure therapy”. Literature inclusion and exclusion criteria are listed in Table 1.

**Table 1:** Literature search inclusion and exclusion criteria ciNPT- closed incision negative pressure therapy (PREVENA™ Incision Management System, KCI, an ACELITY Company, San Antonio, US)

Inclusion Criteria	Exclusion Criteria
Use of ciNPT	Abstract
English language	Review Article
Study population >1	Meta-analysis
	Single case reports
	Non-English article
	Veterinary study
	Non-clinical reports
	Pre-clinical studies
	Use of non-ciNPT device

Results

Literature Search

A total of 88 articles were identified during the literature search. Once duplicates and articles not meeting the inclusion criteria were removed, 40 articles were identified. Of these included articles, 12 were randomized controlled trials (RCTs), six were prospective cohort studies, 15 were retrospective comparative studies, and seven were case series.

The successful application of ciNPT on clean, closed wounds has been reported in a broad spectrum of patients and operative interventions resulting in positive clinical results in a majority of the RCTs identified in the literature search (Table 2) [11-19]. Similarly, rates of SSIs, surgical site complications, readmissions, and/or reoperations were reduced in patients receiving ciNPT compared to historical control populations [20-25].

**Table 2:** Randomized controlled trial evidence reporting the use of closed incision negative pressure therapy *Control groups received traditional surgical dressings; ciNPT - closed incision negative pressure therapy; SSI - surgical site infection; RR - relative risk; CI - confidence interval; NSAID - nonsteroidal anti-inflammatory drug

Author	Patient Population	Results
Engelhardt et al. [26]	132 patients Vascular surgery ciNPT, n=64; *Control, n=68	Infection rates slightly lower in ciNPT patients (9/64 ciNPT vs 19/68 control; p=0.055). Early infection rates were similar between the two groups (4/64 ciNPT vs 10/68 control; p=0.125).
Gombert et al. [11]	204 patients Vascular surgery ciNPT, n=98; *Control, n=90	Significantly lower levels of SSI in ciNPT group (13/98 vs 30/90 control; p=0.0015).
Gunatilake et al. [12]	82 patients Cesarean delivery ciNPT, n=39; *Control, n=43	Reduced surgical site occurrences in ciNPT group (2/39 vs 7/43; p=0.16). Significantly reduced pain at rest (29/39 vs 39/43, p<0.01). Significantly reduced pain with pressure in ciNPT group (25/39 vs 42/43, p<0.001). Significantly reduced total narcotic use in ciNPT group (55.9% vs 79.1%, p=0.036). Similar rates of acetaminophen use in both groups (p=0.47). Similar rates of total NSAID use in both groups (p=0.87).
Javed et al. [13]	123 patients Abdominal surgery ciNPT, n=62; *Control, n=61	Reduced SSI in ciNPT group (9.7%) vs control group (31.1%, RR = 0.31, 95% CI 0.13-0.73; p=0.03). Reduced rate of superficial SSI in ciNPT group (6.5%) vs control group (27.9%; p=0.002). Similar rate of deep SSI in both groups (3.2% vs 3.3%; p=0.99). Similar lengths of stay in the ICU (1 day vs 1 day) and hospital (7 days vs 8 days) for both groups (p>0.05). Similar rates of reoperation for ciNPT and control groups (1.6% vs 6.6%; p=0.21). Reduced rates of readmission for ciNPT group, though not statistically significant. (8.1% vs 19.7%; p=0.07) Similar rates for readmission for SSI between both groups (4.8 vs 9.8; p=0.32).
Kwon et al. [14]	119 incisions Vascular surgery ciNPT, n=59; *Control, n=60	Reduced surgical site occurrences in high-risk ciNPT group (11.9%) vs high-risk control group (26.7%; p<0.01). Reduced reoperation rate in high-risk ciNPT group (8.5%) vs high-risk control group (18.3%; p<0.05). Reduced readmission rate in high-risk ciNPT group (6.8%) vs high-risk control group (16.7%; p<0.04). Similar length of stay in both high-risk groups (10.6 days for both).
Lee et al. [15]	60 patients Cardiac surgery ciNPT, n=33; *Control, n=27	Similar SSI rates in both groups (0/33 vs 1/27 control; p>0.05). ciNPT was tolerated by patients. ciNPT group had a shorter length of stay (6 days vs 10 days control; p=0.008).
Lee et al. [16]	102 patients Vascular surgery ciNPT, n=53; *Control, n=49	Reduced SSI rates in ciNPT group (11% vs 19% control; p=0.24) Significantly shorter length of stay in ciNPT group (6.4 days vs 8.9 days control; p=0.01). Similar rates of readmission (3.8% vs 4.1% control) and reoperation (3.8% vs 2.0%) for SSI between both groups (p>0.05).
Muller-Sloof et al. [17]	51 patients Breast reconstruction surgery ciNPT, n=25; *Control, n=26	Reduced rates of surgical dehiscence in ciNPT group (8% vs 33%; p=0.038). Similar rates of SSI between both groups (4% vs 0%; p>0.05).
Murphy et al. [27]	284 patients Colorectal surgery ciNPT, n=144; *Control, n=140	Similar incidence of SSI at 30-days postoperatively between both groups (32% ciNPT vs 34% control; p=0.66). Similar rates of reoperation between both groups (4% vs 4%; p=0.96). Similar length of stay between both groups (p=0.68).
Newman et al. [18]	160 patients Arthroplasty surgery ciNPT, n=80; *Control, n=80	Wound complication rate was significantly lower in ciNPT group (9/80 vs 22/80 control; p=0.009). Similar rates of readmission between the groups (16/80 vs 16/80; p=0.99). Reduced rates of reoperation in ciNPT group (5/80 vs 11/80; p=0.63).
Pleger et al. [19]	100 patients, 129 incisions Vascular surgery ciNPT, n=58 incisions; *Control, n=71 incisions	Significant reduction in wound complications in ciNPT group (5/58 vs 30/71 control; p<0.0005). Significant reduction in reoperation in ciNPT group (1/58 vs 10/71; p=0.022).
Ruhstaller et al. [28]	136 patients Cesarean delivery ciNPT, n=67; *Control, n=69	Similar rates of wound complications were seen between both groups (4.9% vs 6.9% control; p=0.71).

Economic Analysis of Published Clinical Studies

Only two studies identified from the literature search examined the economic impact of ciNPT use in patients at high risk for developing SSIs (Table 3) [14, 28]. The Kwon et al. study indicated a cost savings of $6,045 in ciNPT patients; however, Ruhstaller et al. found an increase in patient costs ($10,300) in the ciNPT patient group [14, 28]. More economic studies are needed to fully assess the potential economic benefit of ciNPT use.

**Table 3:** Economic evidence in the use of closed incision negative pressure therapy ciNPT - closed incision negative pressure therapy

Author	Patient Population	Results
Kwon et al. [14]	119 incisions; vascular surgery ciNPT, n=59 Control, n=60	Cost for high-risk ciNPT group care was $6,045 less than the high-risk control group, though not statistically significant (p=0.11).
Ruhstaller et al. [28]	136 patients; Cesarean delivery ciNPT, n=67 Control, n=69	The prevention of one SSI would increase patient costs an average of $10,300 (US). 28 ciNPT would need to be placed to prevent one SSI.

Patient Selection

The potential clinical value of ciNPT over clean, closed surgical incisions in a variety of patients at risk for developing surgical site complications has been shown in a growing body of literature. A review the RCT literature reports that patients that benefit most from ciNPT use were those at greater risk for infection, seroma, hematoma, and dehiscence [14-16, 18, 19]. These patients were found to have one or more risk factors that negatively affected wound healing and were undergoing high-risk surgical procedures. Stannard and associates have proposed the use of a Patient Grading System, which may be helpful in identifying candidates for ciNPT use (Table 4) [29]. Known patient risk factors or comorbidities include diabetes, obesity, smoking, hypertension, steroid use, radiation exposure, and other factors affecting wound healing (Table 5) [30, 31]. Patients without pre-existing medical conditions may not be candidates for the ciNPT use as their surgical incisions usually heal well on their own [31, 32].

**Table 4:** Patient grading system *Known risk factors includes diabetes, obesity, tobacco use, hypertension, steroid use, radiation therapy, chemotherapy, and peripheral arterial disease. Adapted from Stannard et al. [29].

Patient Risk Factors	Description	Grade
Otherwise healthy, no pre-existing medical conditions	No risk factors	Grade 1
Presence of a known risk factor*	Single risk factor	Grade 2
Presence of multiple known risk factors	Multiple risk factors	Grade 3

**Table 5:** Patient risk factors for incision complications Adapted from Riou et al. [30] and Abbas et al. [31].

Patient Risk Factors	Wound Risk Factors
Age > 65	Wound infection
Pulmonary disease	Length and depth of incision
Vascular disease	Foreign body in the wound
Hemodynamic instability	Type of injury
Ostomies	
Hypoalbuminemia	
Systemic infection	
Obesity	
Hyperalimentation	
Ascites	
Malignancy	
Hypertension	
Anemia	
Jaundice	
Diabetes (poor control)	
Active tobacco use	
Radiation therapy	
Steroid use	

Discussion

SSIs and other common surgical site complications (dehiscence, hematoma, and seroma formation) can lead to serious and often life-threatening complications. Traditional postoperative incision management has included gauze dressings, adhesive dressings, and skin adhesives; however, ciNPT can offer healthcare providers another incision management option.

A growing body of evidence has reported reduced rates of SSI and other surgical site complications resulting from ciNPT usage. The literature search identified 12 RCTs, a majority of which reported reduced SSI rates, reduced readmission rates, and reduced reoperation rates. Six of the non-RCT, comparative studies identified also reported reduced rates of SSIs, readmissions, and reoperations [20-25]. However, these studies examined a wide range of patients, with a variety of comorbidities, undergoing different surgical procedures. Thus, a definitive conclusion on the potential clinical benefit of ciNPT for specific patient groups or surgical procedure cannot be made with this literature search. Future meta-analyses limited to specific patient groups and surgical procedures are necessary.

Health economic data for ciNPT use is limited. While only two studies were identified in the literature search, they provided differing conclusions [14, 28]. Additionally, since 2009, only three other studies examining the health economics of ciNPT use have been published [33-35]. Chopra et al. [33] report that in their 829 patients undergoing abdominal wall reconstruction, ciNPT use resulted in an estimated cost savings of $1,542.52 and could be a cost-effective option when the estimated SSI rate is above 16% for the patient population. Similarly, Grauhan et al. [34] reported an estimated cost savings of 60,000,000€ to 90,000,000€ per year in Germany for patients undergoing cardiac surgery. Matatov and colleagues [35] noted that for their vascular surgery patients, none required an extended hospital stay or care for SSI, suggesting cost savings with ciNPT use compared to the >$45,000 costs for infection care and extended hospital stay for two control patients with Szilagyi grade III infection. Despite these additional studies, the health economic analysis of ciNPT use requires further research as the current body of literature is too limited to provide a definitive conclusion.

Limitations

This review is not without limitations. The review presented is not a systematic meta-analysis, but a literature review including both RCTs and observational studies and a variety of patient subgroups and surgical types. A number of meta-analyses have been published in recent years with results in favor of ciNPT use; however, they do not list patient use selection recommendations which we believe is beneficial for healthcare providers considering adding ciNPT to their patient treatment plans. As this review included a variety of patients and surgical procedures, additional patient subset or surgical type-specific meta-analyses are necessary to draw conclusions on the clinical effectiveness of ciNPT use. Additionally, health economic data regarding ciNPT use is limited. More research is needed as current data is too limited to provide a definitive conclusion.

## Conclusions

The published literature suggests that patients at high risk for developing a surgical site complication may benefit ciNPT during the immediate postoperative period. Additional studies are needed across various surgical disciplines to further assess the safety, and cost-effectiveness of ciNPT use in patient populations.
